# Optimized Liposomal Delivery of Bortezomib for Advancing Treatment of Multiple Myeloma

**DOI:** 10.3390/pharmaceutics15122674

**Published:** 2023-11-25

**Authors:** Chi Zhang, Jimmy Chun-Tien Kuo, Yirui Huang, Yingwen Hu, Lan Deng, Bryant C. Yung, Xiaobin Zhao, Zhongkun Zhang, Junjie Pan, Yifan Ma, Robert J. Lee

**Affiliations:** 1 Division of Pharmaceutics and Pharmacology, College of Pharmacy, The Ohio State University, Columbus, OH 43210, USA; zhang.9395@osu.edu (C.Z.); kuo.249@osu.edu (J.C.-T.K.); huang.4650@osu.edu (Y.H.); zhang.5763@osu.edu (Z.Z.); 2 The Whiteoak Group, Inc., Rockville, MD 20855, USA; yingwen.hu@thewogroup.com (Y.H.); lan.deng@thewogroup.com (L.D.); bryant.yung@thewogroup.com (B.C.Y.); ben.zhao@thewogroup.com (X.Z.); 3 William G. Lowrie Department of Chemical and Biomolecular Engineering, The Ohio State University, Columbus, OH 43210, USA; pan.687@osu.edu

**Keywords:** Bortezomib, liposome, remote-loading, multiple myeloma, Tiron

## Abstract

Bortezomib (BTZ), a boronic acid-derived proteasome inhibitor, is commonly employed in treating multiple myeloma (MM). However, the applications of BTZ are limited due to its poor stability and low bioavailability. Herein, we develop an optimized liposomal formulation of BTZ (L-BTZ) by employing a remote-loading strategy. This formulation uses Tiron, a divalent anionic catechol derivative, as the internal complexing agent. Compared to earlier BTZ-related formulations, this alternative formulation showed significantly greater stability due to the Tiron–BTZ complex’s higher pH stability and negative charges, compared to the meglumine–BTZ complex. Significantly, the plasma AUC of L-BTZ was found to be 30 times greater than that of free BTZ, suggesting an extended blood circulation duration. In subsequent therapeutic evaluations using two murine xenograft tumor models of MM, the NCI-H929 and OPM2 models showed tumor growth inhibition (TGI) values of 37% and 57%, respectively. In contrast, free BTZ demonstrated TGI values of 17% and 11% in these models. Further, L-BTZ presented enhanced antitumor efficacy in the Hepa1-6 HCC syngeneic model, indicating its potential broader applicability as an antineoplastic agent. These findings suggest that the optimized L-BTZ formulation offers a significant advancement in BTZ delivery, holding substantial promise for clinical investigation in not merely MM, but other cancer types.

## 1. Introduction

Multiple myeloma (MM) is a neoplastic disorder characterized by the abnormal proliferation of a single clone of plasma cells, which is responsible for immunoglobulin production [1]. This abnormal growth predominantly occurs within the bone marrow and has the potential to lead to extensive skeletal damage and impair the function of various organs. The U.S. FDA approved Bortezomib (BTZ) for MM treatment in 2003 [2]. As the first proteasome inhibitor, BTZ is notable for its dipeptidyl boronic acid structure, wherein the boron atom exhibits high specificity and affinity in reversibly binding the catalytic sites of the 20S proteasome, subsequently disrupting the 26S proteasome’s catalytic functions [3]. The commercially available form, VELCADE^®^, is a BTZ–mannitol complex, typically administered intravenously at 1.3 mg/m^2^ either standalone or combined with agents like dexamethasone. Its efficacy and tolerability have made it a cornerstone in MM therapy [4]. Although BTZ has a significant therapeutic effect against MM, it has several drawbacks. Its side effects range from severe peripheral neuropathy to neutropenia [5], mainly induced by the off-targeting effects [6]. Additionally, issues like thrombocytopenia arise from its interference with megakaryocyte maturation [7]. Furthermore, the short circulation time coupled with its narrow therapeutic window poses significant challenges for achieving optimal treatment outcomes, greatly complicating its broader clinical adoption [8].

To address the aforementioned issues, liposomal formulations of BTZ (L-BTZ) have been developed over recent years [9,10]. Liposomes composed of biodegradable and biocompatible phospholipids, alongside cholesterol, play a crucial role in shielding drugs from degradation and clearance, thereby extending the therapeutic drugs’ circulation time within the system and bolstering their pharmacokinetic performance [11,12]. This not only enhances the drugs’ solubility and chemical stability, but also contributes significantly to reducing the toxicity of small molecule drugs in vivo, as reported in previous studies [13,14]. Recent studies have demonstrated that L-BTZ offers extended circulation time and enhanced therapeutic effects, especially in murine models of acute myeloid leukemia (AML), chronic myeloid leukemia (CML), and MM models [15,16,17]. Yet, a major setback for these formulations is their limited stability and shelf-life, constraining their potential for clinical translation. As such, we introduce an advanced L-BTZ formulation that leverages the complexation of BTZ with Tiron. Tiron, a catechol featuring two sulfonyl groups, ensures superior aqueous solubility and minimal membrane permeability [18]. We further assessed the in vitro stability of the BTZ boronic ester complexes within developed liposomes, characterized the properties of L-BTZ, and evaluated its in vivo antitumor efficacy across two MM-specific models (NCI-H929 and OPM-2 xenograft models) and one non-MM model (Hepa1-6 HCC syngeneic model).

## 2. Materials and Methods

### 2.1. Materials

(1,2-distearoyl-sn-glycero-3-phosphocholine) (DSPC) 1,2-distearoyl-sn-glycero-3-phosphoethanolamine-N-[methoxy(polyethylene glycol)-2000] (ammonium salt) (mPEG_2000_-DSPE) and cholesterol were purchased from Avanti Polar Lipids (Alabaster, AL, USA). Bortezomib (BTZ) was purchased from LC Laboratories (Woburn, MA, USA). Tiron, meglumine, mannitol, and glucose were purchased from Sigma-Aldrich (St. Louis, MO, USA). Sepharose CL-4B matrix was purchased from GE Life Sciences (Marlborough, MA, USA). NCI-H929, OPM-2, and Hepa1-6 cells were purchased from ATCC (Manassas, VA, USA). RPMI-1640, DMEM, fetal bovine serum, and penicillin-streptomycin solution were purchased from Gibco/ThermoFisher Scientific (Waltham, MA, USA).

### 2.2. pH-Dependent Release of BTZ from Boronic Ester Complexes with Selected Polyol Agents

The polyol agents were prepared at 50 mM in various buffers at different pH (pH 2–10), including Tiron, meglumine, glucose, and mannitol. The polyol aqueous solutions were then mixed with BTZ chloroform solutions under vortex mixing. The residual BTZ within the organic phase was measured by UV-Vis spectrometry at 270 nm after phase separation, and the complexation equilibrium was obtained.

### 2.3. Preparation of Liposomal BTZ (L-BTZ)

Lipids were dissolved in ethanol and mixed in the following molar ratio of DSPC/cholesterol/mPEG_2000_-DSPE = 60/35/5. Specifically, DSPC, cholesterol, and PEG-DSPE were dissolved in ethanol separately at the concentrations of 100 mg/mL, 25 mg/mL, and 100 mg/mL, respectively (the DSPC must be heated to around 55–60 °C before use because the transition temperature is 55.6 °C). We then mixed 63.24 mg DSPC, 18.72 mg PEG-DSPE, and 18.03 mg cholesterol together for every 10 mg bortezomib preparation of liposomal formulation at a drug lipid ratio of 1:10. The lipid-ethanol solution was then quickly injected into an aqueous solution composed of 200 mM Tiron and 200 mM sodium acetate preheated to 60 °C to form liposomes. The liposomes were subjected to high-pressure extrusion (Northern Lipids, Burnaby, BC, Canada) under high-pressure nitrogen gas through 100 nm polycarbonate membrane at 60 °C for three cycles. The liposomes were then dialyzed against PBS for five cycles at room temperature to remove unencapsulated Trion using Slide-A-Lyzer™ Dialysis Cassettes (10K MWCO, ThermoFisher Scientific). Next, BTZ was added to the suspension and the mixture was incubated at room temperature overnight. The unencapsulated BTZ, through dialysis against PBS, subsequently passes the final L-BTZ product through a 0.45 μm PES syringe filter to achieve sterilization in preparation for the animal study. The L-BTZ suspension was then preserved at 4 °C prior to use.

The concentration of BTZ in the suspension was measured by UV-Vis spectrometry at 270 nm after acidification and organic extraction in chloroform. Since Tiron can exhibit absorbance at 270 nm, the encapsulation efficiency of L-BTZ was determined by size exclusion chromatography (SEC) using a Sepharose CL-4B column. For SEC, 500 μL L-BTZ was processed each time. Particle sizes were measured by DLS using a Nicomp Nano Z3000 Zetasizer (Entegris, Billerica, MA, USA). The drug encapsulation efficiency (EE%) was defined by the following equation: EE% = (total amount of BTZ loaded in the liposomes/total amount of BTZ added) × 100%.

### 2.4. Fourier Transform Infrared (FT-IR) Spectra

Fourier transform infrared (FT-IR) spectra (Nicolet 5700, Thermo, Waltham, MA, USA) of blank liposomes and L-BTZ were scanned in the range of 800–4000 cm^−1^, with subsequent analysis conducted in accordance with methodologies in our prior studies [19,20].

### 2.5. Cryogenic Transmission Electron Microscopy (Cryo-TEM) Analysis

The morphology of L-BTZ was examined on a Thermo Scientific Glacios cryogenic transmission electron microscopy (Cryo-TEM, ThermoFisher Scientific, Hillsboro, OR, USA) device using the methods described previously [21,22]. Specifically, for Cryo-TEM analysis, a 10 μL aliquot of L-BTZ nanoparticles was placed on a specimen grid. After blotting away excess liquid, the grid was immediately plunged into liquid ethane to rapidly form a thin film of amorphous ice using the Vitrobot Mark IV system (ThermoFisher Scientific, Hillsboro, OR, USA). CryoTEM visuals were captured at a designated magnification of 57,000× using the Falcon direct electron detector on the Thermo Scientific™ Glacios™ Cryo-TEM. The equipment was set to operate at an acceleration voltage of 200 kV.

### 2.6. Animal Study of L-BTZ Antitumor Activities

Both NCI-H929 and OPM-2 human multiple myeloma cells were cultured in RPMI-1640 media supplemented with 10% (H929) or 20% (OPM-2) FBS and 1X penicillin-streptomycin under 37 °C with 5% CO_2_ atmosphere. Seven to eight-week-old NOD/SCID female mice were housed in a temperature-controlled pathogen-free room under a 12 h light/12 h dark cycle and fed a normal chow diet. For the NCI-H929 subcutaneous xenograft model, cells growing in the exponential phase were collected and the mice were inoculated at 10 million NCI-H929 cells per mouse on the right flank. For the OPM-2 subcutaneous xenograft model, each mouse was first γ-irradiated (150 rads) one day before inoculation and was then inoculated subcutaneously at the right flank region with 10 million OPM-2 tumor cells in a PBS/Matrigel mixture. The treatments were initiated once the tumor sizes reached 80–90 mm^3^, and the mice were treated twice weekly at doses of 0.3–0.5 mg/kg. The approved institutional animal care and use committee (IACUC) numbers of these two animal studies were AN-2004-12-1453 and AN-2004-12-1963, respectively.

Hepa1-6 murine hepatocellular carcinoma (HCC) cells were cultured in DMEM media supplemented with 10% FBS. Seven to eight-week-old C57BL6 female mice were inoculated at 0.5 million Hepa1-6 cells per mouse on the right flank. The treatments were initiated once the tumor sizes reached 80–90 mm^3^, and the mice were treated twice weekly at doses of 0.5–1.0 mg/kg. The approved IACUC number of this animal study was 2013A00000117-R2.

### 2.7. Pharmacokinetics Analysis

Female mice were treated with 0.6 mg/kg of free BTZ (BTZ–mannitol complex aqueous solution) or L-BTZ through a single tail-IV bolus injection. Blood samples were collected at various time points and the plasma BTZ concentrations were analyzed using a Waters UPLC/Triple QUAD 6500 LC-MS/MS system, with an ACQUITY UPLC^®^ BEH C18 1.7 µm, 2.1 × 50 mm column. Standard solutions were prepared and diluted in a 50/50 (*v*/*v*) methanol/water mixture at 5–5000 ng/mL. To analyze the plasma BTZ concentrations, 20 µL of plasma sample was mixed with 20 µL hydrochloric acid (1M) on the vortex, and the plasma proteins were precipitated by mixing the plasma solution with 280 µL methanol containing internal standard (Propranolol). The plasma protein precipitation was removed by centrifugation at 4000 rpm for 20 min at 4 °C. Next, 100 µL of supernatant was diluted with 300 µL 0.1% formic acid prior to the injection into the LC-MS/MS system. The MP-A was 0.1% formic acid in water, and MP-B was formic acid in acetonitrile. The wash solvents were 200 μL MeOH: ACN: Isopropyl Alcohol: H_2_O = 3:3:3:1 (strong wash), and 600 µL 30% MeOH in H2O (weak wash). The injection volume was 2 μL. Flow rate was 600 µL/min. The gradient step was 80% MP-A (20% MP-B) firstly, followed by 5% MP-A (95% MP-B).

The linear regression equation as a calibration curve was obtained by regressing calculation using weighted least squares, and the ordinate (y) was the peak area ratio of the concentration of the compound and the internal standard. The integral for BTZ and the internal standard peak area was computed using Analyst 1.7.1 data processing software.

### 2.8. Statistical Analysis

To compare tumor volumes of different groups on a pre-specified day, Bartlett’s test was used to test the assumption of homogeneity of variance across all groups. When the *p*-value of Bartlett’s test was ≥ 0.05, we ran a one-way ANOVA to test the overall equality of means across all groups. If the p-value of the one-way ANOVA was <0.05, we further performed post hoc testing by running Tukey’s HSD tests for all pairwise comparisons and Dunnett’s tests for comparing each treatment group with the vehicle group. When the *p*-value of Bartlett’s test was <0.05, we ran a Kruskal–Wallis test to test the overall equality of medians among all groups. If the *p*-value of the Kruskal–Wallis test was <0.05, we further performed post hoc testing by running Conover’s non-parametric test for all pairwise comparisons or for comparing each treatment group with the vehicle group, both with single-step *p*-value adjustment. All statistical analyses were done in R (version 3.3.1). All tests were two-sided unless otherwise specified, and *p*-values of <0.05 were regarded as statistically significant.

For survival analysis, the survival time was analyzed by the Kaplan–Meier method. The survival time was defined as the time from the day of randomization until animal death or ethical endpoint. For each group, the median survival time (MST) and the increase in life span (ILS) were calculated. The Kaplan–Meier curves were also constructed for each group, and the log-rank test was used to compare survival curves between groups.

## 3. Results

Bortezomib (BTZ) is a potent proteasome inhibitor that is effective in treating multiple myeloma (MM). However, due to its short circulation time and narrow therapeutic window, some liposomal formulations were developed [23]. Despite the attempts to encapsulate BTZ into liposomes by various strategies, its encapsulation efficiency and stability remain problematic [24]. “BTZ has demonstrated a reversible reaction with polyols, including meglumine, mannitol, and glucose, producing cyclic ester prodrugs [9]. However, the stability of these boronic ester prodrugs is insufficient for consistent retention within liposomes, resulting in undesirable delivery and therapeutic efficacy. Consequently, there is a pressing need for a better BTZ formulation to enhance MM treatment.

### 3.1. Physical Characterization of Liposomal BTZ (L-BTZ)

**Figure 1:**
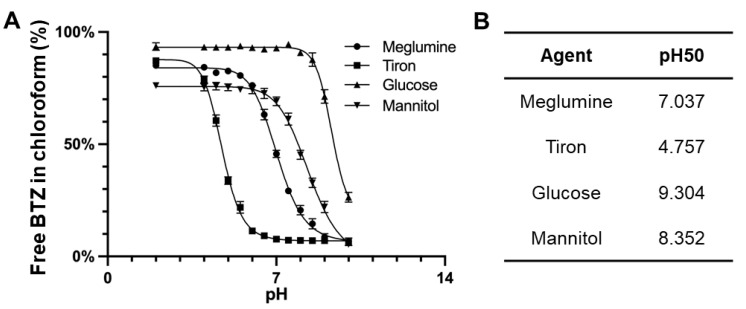
(**A**) pH-dependent dissociation of BTZ from various complexing agents including meglumine, Tiron, glucose, and mannitol. Free BTZ was partitioned into the chloroform phase. (**B**) pH50 was analyzed using four-parameter sigmoidal curves based on the pH-dependent dissociation results.

Boronic acids, under basic conditions, commonly form boronic esters with diols, resulting in tetrahedral anions. First, we evaluated the stability of BTZ boronic ester complexes using four different complexing agents across various pH conditions. Given BTZ’s hydrophobic nature and limited aqueous solubility, we utilized chloroform to extract BTZ released from these esters. As shown in Figure 1, our findings suggested a clear pH-dependent stability for the BTZ complex, with BTZ release from boronic esters being most prevalent at lower pH levels. Among these four agents tested, Tiron was found to maintain boronic ester complexation at reduced pH levels, indicating its strong affinity for BTZ. Notably, the pH50 of Tiron (4.748) was significantly lower than that of meglumine (7.034), the next most effective ligand for BTZ. This superior performance of Tiron can be attributed to the favorable bond angles of the catechol hydroxyl elements, which orient the two hydroxyls on a singular plane, giving Tiron an edge in BTZ complexation stability over other polyols like meglumine, mannitol, and glucose (Table 1).

**Figure 2:**
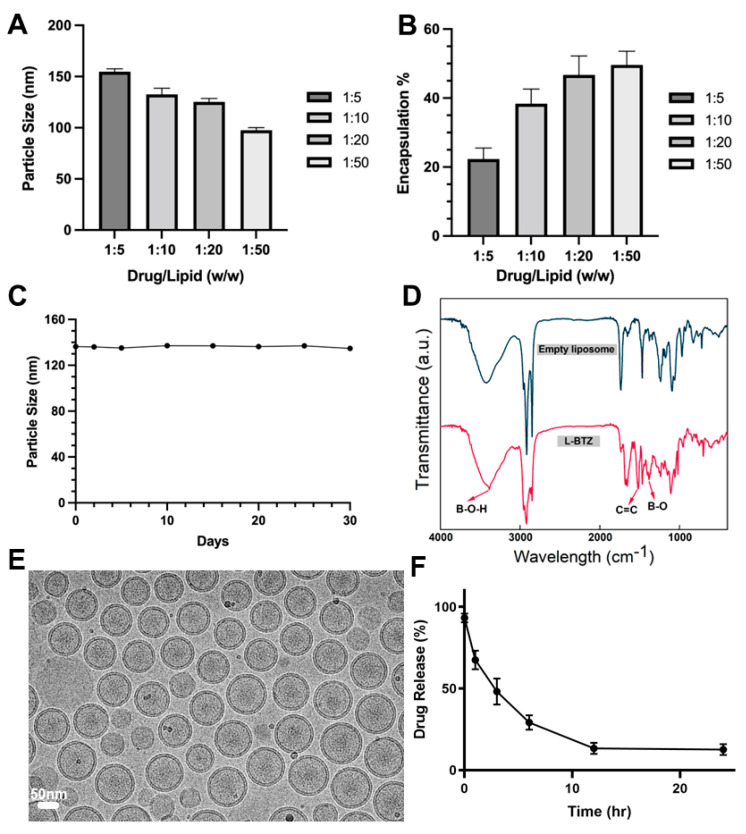
Particle size (**A**) and drug loading efficiency (**B**) of L-BTZ with varied drug-to-lipid ratios. (**C**) Measurement of particle size variations during the long-term storage. The results suggest L-BTZ remains stable at 4 °C for a period over 30 days. (**D**) Fourier-transform infrared spectroscopy (FTIR) of empty liposomes and L-BTZ. (**E**) Cryogenic transmission electron microscopy (cryo-TEM) analysis of the L-BTZ particles. Scale bar: 50 nm. (**F**) In vitro BTZ release profile in PBS at pH 6.0 for 24 h.

For a more detailed characterization of L-BTZ, we then analyzed its particle sizes (Figure 2A) and drug loading efficiencies (Figure 2B) across different drug-to-lipid (DL) ratios. The mean particle size increased from 99.4 nm to 152.0 nm when the drug lipid ratio changed from 1:50 to 1:5, while the drug loading efficiency decreased as the DL ratio increased. When comparing L-BTZ’s drug loading efficiency across DL ratios of 1:10, 1:20, and 1:50, we observed no substantial disparities. From a cost-effectiveness perspective, we opted for the 1:10 DL ratio for the following experiments. To evaluate the storage stability of L-BTZ, we monitored its particle sizes over a 30-day span, storing the samples at 4 °C (Figure 2C). Throughout this duration, the particle size remained consistent, suggesting the robust stability of the L-BTZ formulation. Then, we assessed the drug release pattern, distinguishing the encapsulated BTZ within the liposomes from the free BTZ, using a Sepharose CL-4B size exclusion column (SEC). Obviously, there was no significant drug leak during storage throughout the tested period. Then, Fourier-transform infrared spectroscopy (FTIR) analyses (Figure 2D) were performed using KBr tablet methods. Compared to empty liposomes, the characteristic peaks of BTZ could be found around 1376 cm^−1^, 3387 cm^−1^, and 1516 cm^−1^, which corresponds to B–O stretching and O–H stretching in the B–O–H group, and C=C aromatic stretching in the BTZ structure, respectively. These characteristic peaks of BTZ suggested successful encapsulation of BTZ in liposomes. In addition, the analysis of cryogenic transmission electron microscopy (cryo-TEM) indicates L-BTZ’s spherical morphology and suggests its size consistency and integrity (Figure 2E). Lastly, the BTZ release profile was investigated in PBS at pH 6.0 for the simulation of the tumor microenvironment. After 3-h incubation, over 50% of the encapsulated LBZ was released (Figure 2F).

### 3.2. In Vivo Antitumor Efficacy Evaluation of L-BTZ in Human Multiple Myeloma (MM) Xenograft Models

**Figure 3:**
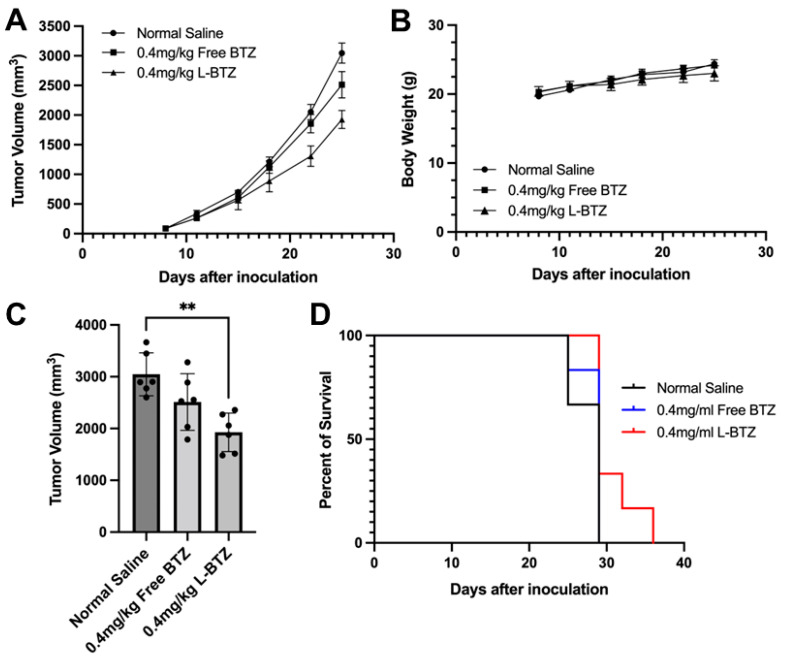
In vivo antitumor efficacy of L-BTZ in the NCI-H929 xenograft model. (**A**) Tumor volume of mice treated with normal saline, 0.4 mg/kg free BTZ, and 0.4 mg/kg L-BTZ. (**B**) Mean body weight of each treatment group throughout the treatment period. (**C**) Average tumor volume on day 25. ** *p* = 0.0017 for the 0.4 mg/kg L-BTZ treatment group when compared to normal saline cohort. (**D**) Kaplan–Meier survival curves.

To assess the antitumor efficacy of L-BTZ against multiple myeloma (MM), we first used NCI-H929 subcutaneous tumor-bearing mice. These mice were treated with either free BTZ (BTZ–mannitol complex, or free BTZ) or L-BTZ, both at 0.4 mg/kg, through tail IV injections twice weekly for three weeks. As shown in Figure 3A, there was no significant difference in tumor sizes between the mice given normal saline and those administered with free BTZ (*p* = 0.16). Notably, the tumors of mice treated with L-BTZ were significantly smaller compared with those treated with free BTZ, indicating the superior effect of L-BTZ in inhibiting tumor growth (Figure 3C). Quantitively, the tumor growth inhibition (TGI) recorded was 18% for the free BTZ group, while it reached 37% for the L-BTZ group. In terms of safety metrics, all the treatment groups showed no significant difference in body weight loss (Figure 3B), suggesting minimal systemic toxicity for both BTZ-related cohorts at the 0.4 mg/kg dosage. Consistently, the median survival time (MST) for each of the three groups was at 29 days (Figure 3D).

**Figure 4:**
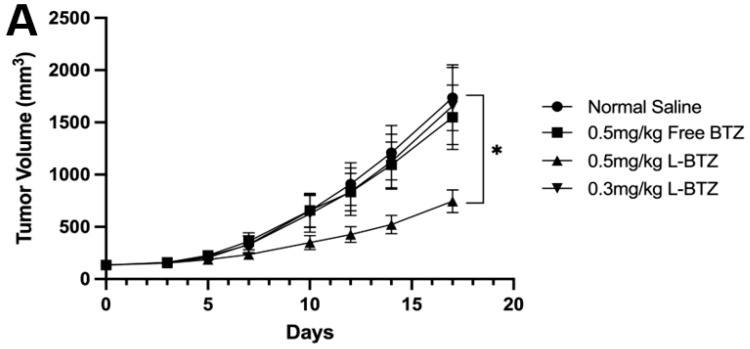

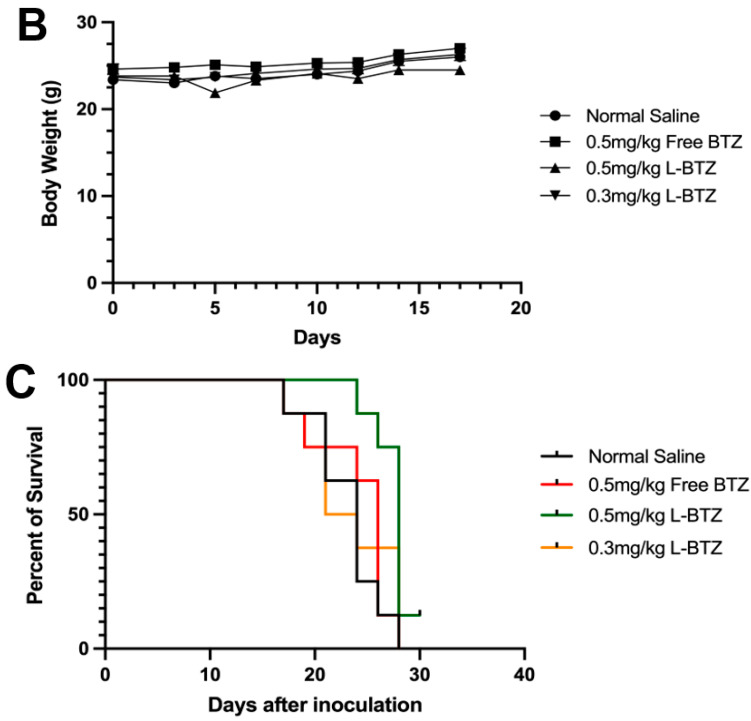
In vivo antitumor efficacy of L-BTZ in the OPM-2 xenograft model. (**A**) Tumor volume of mice treated with normal saline, 0.5 mg/kg free BTZ, 0.5 mg/kg L-BTZ, and 0.3 mg/kg L-BTZ. (**B**) Mean body weight of each treatment group throughout the treatment period. (**C**) Kaplan–Meier survival curves. * *p* < 0.05.

To further validate the antitumor efficacy of L-BTZ in MM treatment, especially in BTZ-resistant scenarios, the OPM-2 xenograft model, another human MM cell line resistant to BTZ compared to NCI-H929, was applied [25]. In Figure 4A, mice treated with free BTZ at a dosage of 0.5 mg/kg exhibited only a minimal antitumor effect against the OPM-2 tumors with a TGI of 10.79%. Also, there was no significant difference observed between the control group and the free BTZ group. In contrast, L-BTZ at the same dosage (0.5 mg/kg) presented a remarkable antitumor impact against OPM-2 tumors, achieving a TGI of 57.13%. However, when the dosage was reduced to 0.3 mg/kg, L-BTZ’s antitumor efficacy diminished significantly. This suggests that dosages ≤0.3 mg/kg of L-BTZ offer little therapeutic value against the OPM-2 model, highlighting the restricted therapeutic window of BTZ. As shown in Figure 4B, after treatment, there was a moderate decrease in body weight, which the mice managed to recover after being provided with a diet gel. A summarized analysis of tumor growth inhibition across different groups on day 17 following treatment of the subcutaneous OPM-2 human MM xenograft model in NOD/SCID mice can be found in Table 1. The survival curves of the OPM-2 models are shown in Figure 4C. It is worth noting that no mice were euthanized due to substantial weight loss or any adverse effects attributed to drug toxicity. The median survival duration for L-BTZ dosed at 0.5 mg/kg was 28 days, marking a 17% lifespan enhancement with a statistical significance of *p* < 0.01. These outcomes point towards the superior therapeutic benefits of 0.5 mg/kg L-BTZ against MM models compared to other examined groups, suggesting its potential to extend survival durations.

### 3.3. Therapeutic Efficacy of L-BTZ against a Non-MM Model on C57BL/6 Mice

**Figure 5:**
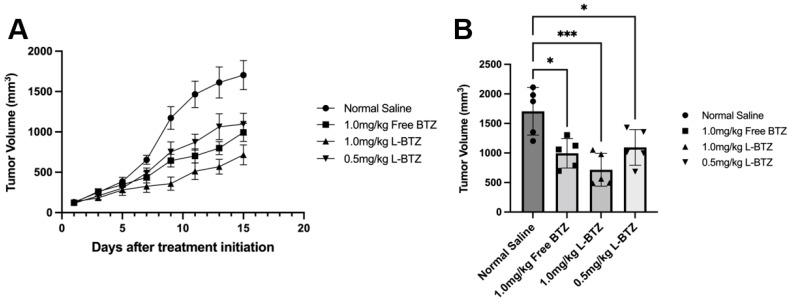
In vivo antitumor efficacy of L-BTZ in the Hepa1-6 syngeneic model. (**A**). Tumor volume of mice treated with normal saline, 1.0 mg/kg free BTZ, 1.0 mg/kg L-BTZ, and 0.5 mg/kg L-BTZ, (**B**)**.** Average tumor volume on day 15. * *p* < 0.05, *** *p* < 0.001.

To explore the potential of L-BTZ as a general antineoplastic agent, the antitumor efficacy of L-BTZ was carried out on a Hepa1-6 HCC syngeneic model on C57BL/6 mice. Randomized mice were treated with either normal saline (as the control), 1.0 mg/kg in-house VELCADE^®^ (free BTZ), 1.0 mg/kg L-BTZ, or 0.5 mg/kg L-BTZ intravenously once every three days for 5 doses. The tumor growth curves were shown in Figure 5, and the TGI of 1.0 mg/kg free BTZ, 1.0 mg/kg L-BTZ, and 0.5 mg/kg L-BTZ treatment groups (compared with the control) were 41.7% (*p* = 0.012), 58.0% (*p* = 0.007), and 35.7% (*p* = 0.033), respectively (Table 2). The result suggested that L-BTZ worked better than free BTZ at the same doses (1.0 mg/kg) and the efficacy was improved at a higher dose, within the tolerated toxicity region. Therefore, the therapeutic outcomes in the Hepa1-6 syngeneic model suggested that the liposomal BTZ has improved therapeutic efficacy in vivo compared to the free drug and has the potential to work as a broad spectrum anti-neoplastic agent not only against hematopoietic malignancies but also solid tumors.

### 3.4. Plasma Clearance Kinetics of L-BTZ

**Figure 6:**
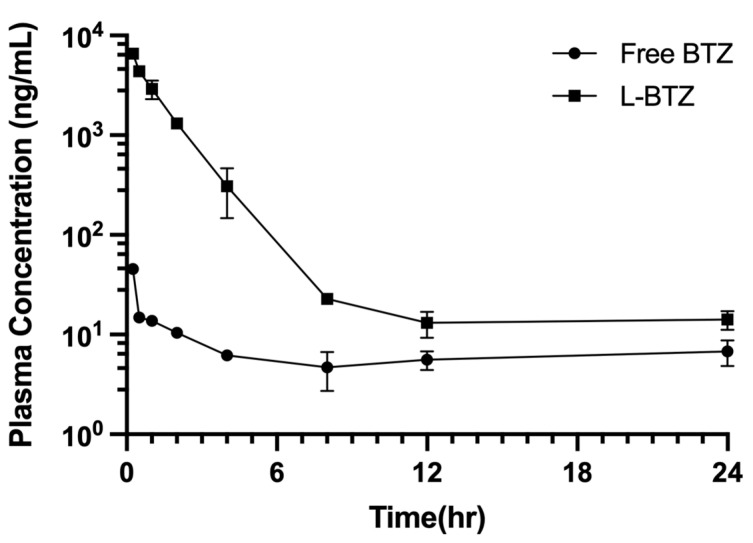
Plasma time concentration profile of free BTZ and L-BTZ in ICR mice. Each mouse was treated with 0.6 mg/kg of BTZ via IV injection.

The pharmacokinetic performance of L-BTZ was examined in vivo and the comparison between the free drug and the liposomal formulation was done to elucidate the plasma concentration alternation of BTZ within the systemic circulation. A single dose of free BTZ or liposomal BTZ at 0.6 mg/kg was given to CD-1 mice via IV injection, and the plasma samples were collected at 0.25, 0.5, 1, 2, 4, 8, 12, and 24 h (Figure 6). The AUC_0–t_ of L-BTZ was ~33 times larger than that of free BTZ, indicating that the liposomal formulation could retain the drug substance within the circulation by reducing clearance through renal filtration, therefore prolonging the circulation time as well as sustained release of active substances, which could maintain the therapeutic concentration within the systemic circulation and further lead to the increase in therapeutic effect (Table 3).

## 4. Discussion

Bortezomib (BTZ), as a proteasome inhibitor, is effective in the treatment of multiple myeloma (MM) and mantle cell lymphoma. Nonetheless, the clinical use of BTZ is hindered by the poor pharmacokinetic profile and off-targeting adverse effects. In pursuit of addressing these limitations, nanotechnology, particularly the use of liposomes, has been employed to restructure BTZ into a stable and preferred drug formulation. In this context, recent research endeavors have reformulated BTZ using liposomal techniques, including surface conjugation or encapsulation [10], and these liposomal formulations were evaluated in various cancer models, such as colon cancer, melanoma [26], or neuroblastoma [27], to determine the broader therapeutic potential of L-BTZ. However, these formulations using mannitol, meglumine, PVA (poly vinyl alcohol), or sorbitol as entrapping agents encountered stability challenges, because both the complexing agent and the BTZ would gradually leach out of the liposomes [17]. While increasing the stability of boronic esters is feasible at elevated pH levels, such an environment can catalyze lipid hydrolysis, resulting in the loss of liposome stability. Given these challenges, there is a clear need for a complexing agent that not only binds strongly to BTZ at near-neutral pH levels but also exhibits minimal permeability compared to the monosaccharide derivatives found in earlier L-BTZ versions. Tiron, due to its aromatic hydroxyls, has a high affinity for BTZ at mildly basic pH levels. Simultaneously, Tiron’s dual sulfonate groups, which are permanently charged, offer the added benefit of minimizing membrane permeability. These features are crucial to maintaining the Tiron concentration gradient and the long-term stability of L-BTZ.

The high affinity between BTZ and catechol derivatives has been recognized previously. Agents such as Tiron [28] and EGCG [29] have been shown to chemically sequester BTZ, diminishing its pharmacological activities. This could be explained by the BTZ complexes not being able to penetrate the cell membrane and being cleared from circulation through excretion. However, our Tiron-based L-BTZ showed potent cytotoxicity in vivo and antitumor efficacy in vivo. This could be due to the retention of Tiron inside the liposomes, thus maintaining high plasma concentrations, while BTZ was released in its boronic acid form in response to the external environment at the site of the tumor. Tiron was not released due to its two negative charges, thus would not sequester BTZ and prevent membrane transport. Increased release of BTZ could be triggered by the increased proton concentration. The phenomenon suggested that the Tiron–BTZ-based liposomal drug could demonstrate high stability under physiological pH during storage and could release the drug through lipid membranes at the acidic environment, such as the tumor microenvironment (TME), to act against cancer cells. The L-BTZ was tested for long-term stability (drug retention) and the result indicated that more than 90% of BTZ remained entrapped in liposomes after 90 days at 4 °C. The mean particle sizes obtained by DLS were not significantly altered during the long-term storage, suggesting that the formulation presented high colloidal stability.

The novel Tiron-based L-BTZ revealed improved therapeutic effects against not only hematological malignancies, but also other carcinomas (HCC in this study) and prolonged plasma circulation time and retention compared to free BTZ (mannitol–BTZ complexes) at the same dose. L-BTZ demonstrated high antitumor efficacy in the NCI-H929 xenograft model at 0.4 mg/kg, with a TGI of 37%, compared with that of free BTZ at 18%. No significant body weight loss was observed during the study, indicating the moderate toxicity of L-BTZ.

Further evaluations of L-BTZ antitumor efficacies on different animal models were done to investigate the potential of applying Tiron-based L-BTZ to other indications. The OPM-2 human MM xenograft model and Hepa1-6 murine HCC syngeneic model were selected, and preferable outcomes were shown on the OPM-2 model due to the resistance to BTZ. The tumors of mice treated with 0.5 mg/kg L-BTZ had slower progression compared to those treated with normal saline or free BTZ at the same dose. The drug resistance of OPM-2 was illustrated by the minor TGI after 0.5 mg/kg free BTZ treatment (TGI ~10%) compared with the control group. On the other hand, 0.5 mg/kg L-BTZ gave moderate, meaningful TGI of ~57%, which demonstrated the exclusive properties using liposomal formulations. However, L-BTZ at 0.3 mg/kg had no therapeutic effect on the OPM-2 model, owing to the narrow therapeutic window of BTZ and insufficient BTZ being released at the tumor sites. Moreover, substantial body weight loss occurred in mice of the 0.5 mg/kg L-BTZ treatment group after the second dose, indicating the cumulative toxicity of BTZ generated by the liposomal formulation, which might be an outcome of prolonged retention shown in the PK study. Furthermore, the superior therapeutic effect of L-BTZ over free BTZ was demonstrated in the Hepa1-6 syngeneic model, and the result further confirmed the potential of the Tiron-based L-BTZ in treating other cancers.

The basic pharmacokinetic profile of Tiron-based L-BTZ was evaluated in this study, and the result demonstrated that the liposomal formulation could increase the plasma AUC significantly by reducing the clearance rate, which further prolonged the circulation time of BTZ in vivo. The AUC was substantially higher compared to L-BTZ based on meglumine reported recently in the literature, possibly due to the increased stability of the Tiron-based L-BTZ. Since BTZ has a narrow therapeutic window, it is essential that the plasma concentration of BTZ should maintain above the minimum effective level for longer time. As mentioned above, the increase in drug retention might induce accumulative toxicity of BTZ in repetitive dosing at higher doses. The body weight loss after dosing of either free BTZ or L-BTZ was observed, proving the narrow therapeutic window of BTZ with a very limited region of concentration between the minimum effective dose and the maximum tolerated dose.

Liposomes are known to preferentially accumulate in the bone marrow and be taken up by phagocytic cells such as osteoclasts, which are critical to both disease progression and bone destruction in MM. The advantages of L-BTZ cannot be fully demonstrated in subcutaneous models used in the current study. Future studies should focus on the interaction between L-BTZ and macrophages and osteoclasts as a potential mechanism for improved therapeutic potential.

In summary, two promising findings were mentioned in this article. First, apart from the BTZ-sensitive NCI-H929 xenograft model, the antitumor activity of L-BTZ was also proven to outperform free BTZ in the BTZ-resistant OPM-2 xenograft model, revealing the promising therapeutic potential of BTZ as a treatment for patients experiencing drug resistance to the current commercially available BTZ. Second, the success of L-BTZ in treating the murine HCC syngeneic model suggested that L-BTZ may be used as a treatment for HCC, not limited to MM. Further investigations are warranted to assess the antitumor properties of Tiron-based L-BTZ.

## Figures and Tables

**Table 1:** Antitumor effect of L-BTZ in OPM-2 xenograft model on day 17 after treatment initiation.

Treatment Group	Tumor Size (mm^3^) Mean ± SEM	T/C * (%)	TGI * (%)	*p*-Value ^a^	MST * (Days)	ILS * (%)	*p*-Value ^b^
Normal Saline	1736.51 ± 314.77	-	-	-	24	-	-
Free BTZ, 0.5 mg/kg	1549.21 ± 308.56	89.21	10.79	0.899	26	8	0.428
L-BTZ, 0.5 mg/kg	744.42 ± 109.04	42.87	57.13	0.019	28	17	0.006
L-BTZ, 0.3 mg/kg	1658.03 ± 368.92	95.48	4.52	0.887	21	−13	0.635

^a^: Compared with the normal saline group on Day 17. ^b^: Compared with the normal saline group using Log-Rank test. *: T/C: tumor size ratio of the treated group to the control group; TGI: tumor growth inhibition rate; MST: mean survival time; ILS: increased life span.

**Table 2:** Antitumor effect of L-BTZ in Hepa1-6 syngeneic model on day 17 after treatment initiation.

Treatment Group	Tumor Size (mm^3^) Mean ± SEM	T/C * (%)	TGI * (%)	*p*-Value
Normal Saline	1704.2 ± 179.78	-	-	-
Free BTZ, 1.0 mg/kg	993.6 ± 111.47	58.3	41.7	0.0118
L-BTZ, 1.0 mg/kg	716.0 ± 123.91	42.0	58.0	0.0007
L-BTZ, 0.5 mg/kg	1095.0 ± 135.23	64.3	35.7	0.0329

*: T/C: tumor size ratio of the treated group to the control group; TGI: tumor growth inhibition rate.

**Table 3:** Pharmacokinetic properties of free BTZ and L-BTZ-treated mice. BTZ was given at 0.6 mg/kg intravenously.

	0.6 mg/kg Free BTZ	0.6 mg/kg L-BTZ
AUC_0−t_ (ng × h/mL)	183	9895
AUC_0−∞_ (ng × h/mL)	318	9947
CLss/F ^a^ (mL/h/kg)	1889	60
Vz/F ^b^ (mL/kg)	37,517	220

^a^: apparent total body clearance (at steady state). ^b^: apparent volume of distribution.

## Data Availability

The data that support the findings of this study are available from the corresponding author upon reasonable request.
